# The Spleen Revisited: An Overview on Magnetic Resonance Imaging

**DOI:** 10.1155/2013/219297

**Published:** 2013-11-25

**Authors:** João Palas, António P. Matos, Miguel Ramalho

**Affiliations:** Radiology Department, Hospital Garcia de Orta, 2801-951 Almada, Portugal

## Abstract

Despite being well visualized by different cross-sectional imaging techniques, the spleen is many times overlooked during the abdominal examination. The major reason is the low frequency of splenic abnormalities, the majority consisting of incidental findings. There has been a steady increase in the number of performed abdominal magnetic resonance imaging (MRI) studies; therefore, it is important to be familiar to the major MRI characteristics of disease processes involving the spleen, in order to interpret the findings correctly, reaching whenever possible the appropriate diagnosis. The spleen may be involved in several pathologic conditions like congenital diseases, trauma, inflammation, vascular disorders and hematologic disorders, benign and malignant tumors, and other disease processes that focally or diffusely affect the spleen. This paper presents a description and representative MRI images for many of these disorders.

## 1. Introduction

Splenic disease has always been a challenge to the radiologist. Despite of being well visualized by different cross sectional imaging techniques, the spleen is many times overlooked during the abdominal examination. The major reason is the low frequency of splenic abnormalities, the majority consisting of incidental findings.

There has been a steady increase in the number of performed abdominal magnetic resonance imaging (MRI) studies; therefore, it is important to be familiar to the major MR imaging characteristics of disease processes involving the spleen, in order to interpret the findings correctly, reaching whenever possible the appropriate diagnosis. Nowadays, MRI permits the characterization of the most common splenic lesions, such as cysts, small hemangiomas, and hamartomas, and improvement in the detection of malignant diseases such as lymphoma and metastases [1].

The spleen may be involved by several pathologic conditions including congenital diseases, trauma, inflammation, vascular disorders and hematologic disorders, benign and malignant tumors, and other disease processes that focally or diffusely affects the spleen. This article presents a description and representative MRI images for many of these disorders.

## 2. The Normal Spleen

The spleen is located in the left hypogastric quadrant of the abdomen and is fixed in its intraperitoneal position beneath the 9th to 11th intercostal spaces by the splenorenal, splenocolic, splenogastric, and phrenicosplenic ligaments. The configuration of the spleen is variable (typically coffee bean shaped), as well as the size, which is related with the patient's morphological type and age [2]. The organ's convex face lies adjacent to the diaphragm. The concave side of the spleen has contact with the stomach, left kidney, and colon flexure. The splenic hilum is found within this concavity and acts as an entry and exit route for the arterial, venous, and lymphatic vessels and nerves. The spleen is divided into two compartments, namely, the red and white pulp, separated by the marginal zone. The white pulp is made up of T- and B-lymphocytes and is located centrally, while the red pulp is composed of rich plexuses of tortuous venous sinuses [3].

**Figure 1:**
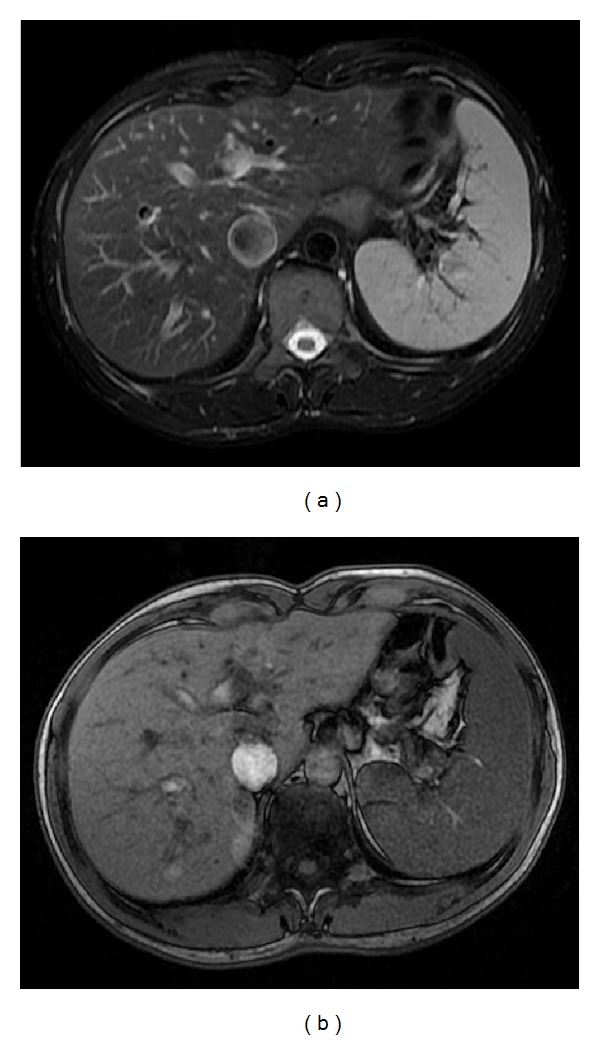
The normal spleen. Axial T2wi FSE with fat suppression (a) and axial T1wi out-of-phase GRE (b). The spleen shows the typical coffee bean configuration. On T1wi (b), the normal signal intensity of the spleen is lower than that of hepatic tissue. Conversely, on T2wi, the spleen shows higher signal intensity.

On T1-weighted MR images, the normal signal intensity of the spleen is lower than that of hepatic tissue. Conversely, on T2-weighted images, the spleen shows higher signal intensity, appearing brighter than the liver [4] (Figure 1). The distinctive microscopic anatomy of the spleen may be reflected on diffusion-weighted images (DWI) and ADC maps. Prior studies have shown significant differences in the mean ADCs between the spleen and other abdominal organs [5, 6].

**Figure 2:**
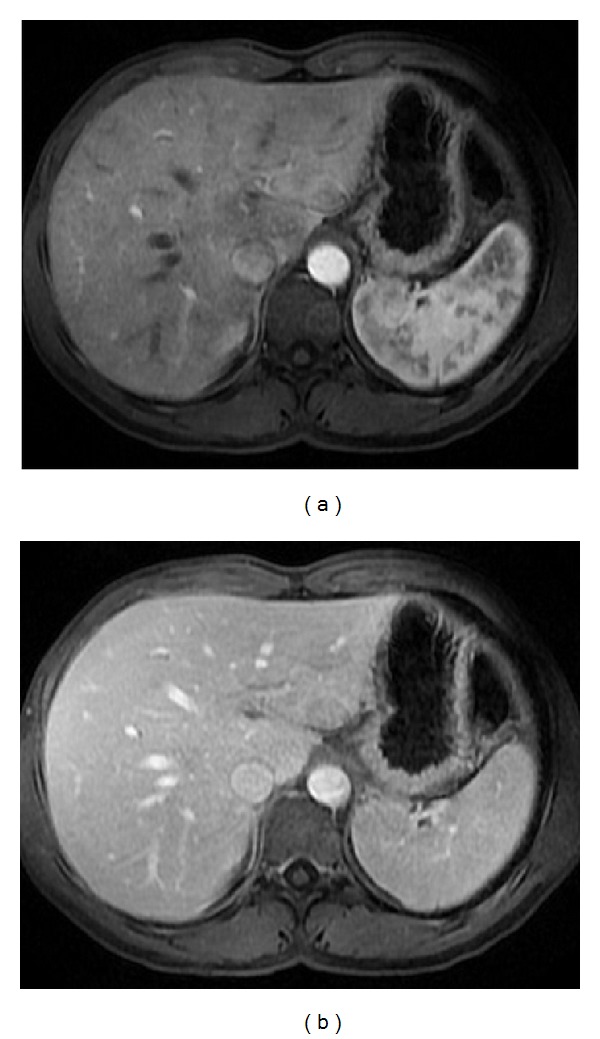
Pattern of enhancement of the normal spleen. Postcontrast axial T1wi 3D-GRE with fat suppression in the arterial (a) and venous (b) phase. The spleen shows a heterogeneous pattern enhancement immediately after contrast material administration (a), secondary to differences in flow between the red and white pulps, becoming homogeneous in venous (b) and interstitial phases.

The spleen demonstrates a heterogeneous serpentine or arciform pattern enhancement immediately after contrast material administration, secondary to differences in flow between the red and white pulps, becoming homogeneous in venous and interstitial phases [4] (Figure 2). Any heterogeneity after this period is considered pathologic [7, 8].

## 3. Congenital Diseases and Normal Variants

The congenital absence of spleen is known as asplenia and the presence of one or more spleens is known as polysplenia. Both are very rare and usually associated with other congenital abnormalities.

**Figure 3:**
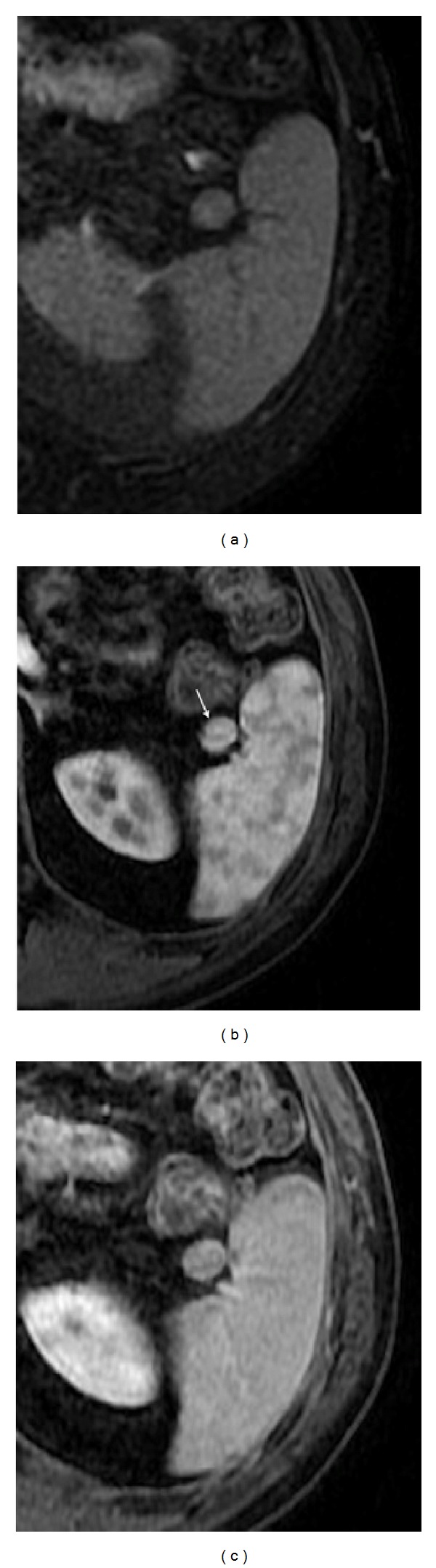
Accessory spleen. Axial T2wi FSE with fat suppression (a) and postcontrast axial 3D-GRE T1wi with fat suppression images at the arterial (b) and venous (c) phases. An accessory spleen is shown near the splenic hilum. Note the similar signal intensity and dynamic behavior comparable with the splenic parenchyma.

The accessory spleens may be found in 10% of the population [9], more frequently in women, usually with less than 4 cm in size and located near the splenic hilum or near the pancreatic tail (Figure 3). More than two accessory spleens occur in less than 5%. The presence of accessory splenules may arise within the substance of solid organs, notably the pancreas [10]. The presence of a well-marginated rounded mass located within 3 cm of the distal tail of the pancreas with signal intensity features of the spleen on all MR sequences suggests the diagnosis of intrapancreatic accessory spleen (IPAS) [11]. However, other entities may mimic the signal intensity and postgadolinium enhancement features of IPAS. Therefore, DWI and SPIO-enhanced MRI can be used to characterize the lesion and to establish the definite diagnosis [3].

**Figure 4:**
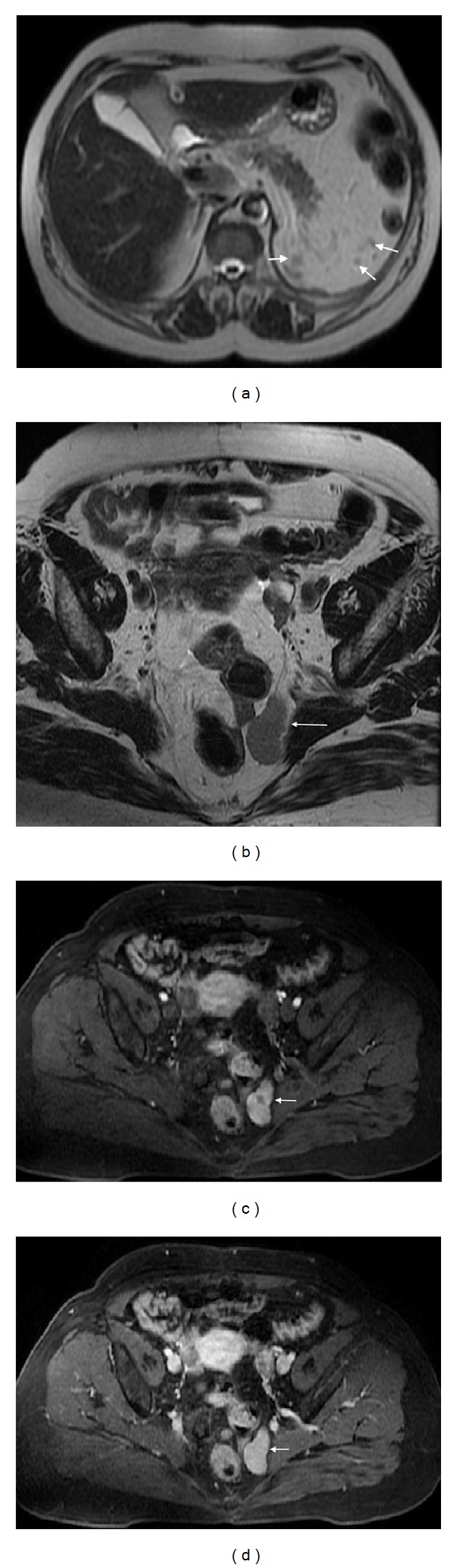
Pelvic splenosis. Axial (a) T2wi SSFSE, axial (b) T2 wi FSE and postcontrast axial T1wi 3D-GRE with fat suppression in the arterial (c) and venous (d) phase. This patient with history of splenectomy following a car accident underwent pelvic MRI to clarify pelvic masses depicted in previous CT. There are multiple nodular masses in the left hypochondrium (arrows, (a)) consistent with splenosis. There are also multiple well-defined nodules in the pelvis demonstrating high signal intensity on T2wi (arrow, (b)), with heterogeneous enhancement immediately after contrast material administration (c), becoming homogeneous in the venous (d) phase, consistent with splenosis.

Splenosis develops when splenic tissue is seeded within the abdomen or pelvis (Figure 4) following trauma [12]. 

**Figure 5:**
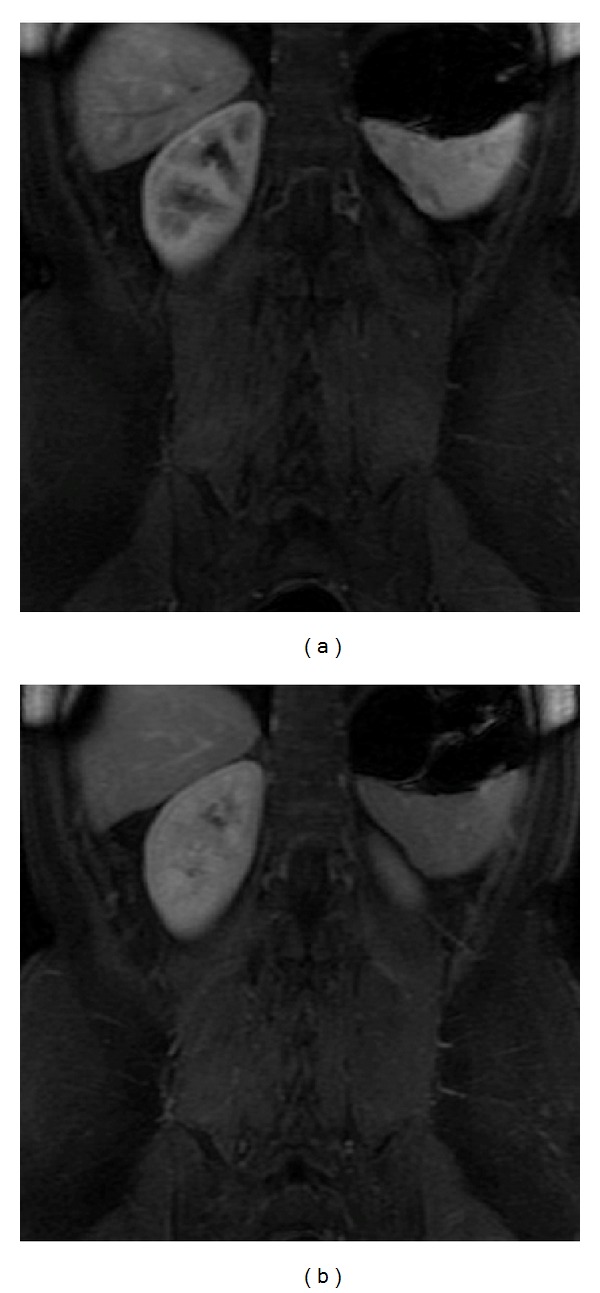
Upside-down spleen. Coronal postcontrast 3D-GRE T1wi with fat suppression at the arterial (a) and venous (b) phases. An abnormal splenic rotation is seen. The hilum is superiorly located and the convex border is adjacent to the left kidney.

The “upside-down spleen” is a normal variant due to an abnormal splenic rotation where the hilum is superiorly located and the convex border is medial and adjacent to the left kidney [13] (Figure 5).

All these normal variants and congenital abnormalities are usually easy to recognize.

## 4. Inflammatory/Infectious Diseases

### 4.1. Abscesses

Splenic abscess is an uncommon lesion with high mortality rates, because of delayed detection and treatment [14, 15]. The frequency of splenic abscess has recently increased due to the higher number of immunocompromised patients, as those with hematologic disorders (e.g., leukemia), those with recreational intravenous drug abuse, and those with acquired immunodeficiency syndrome (AIDS). These lesions may be single or multiple [7].

**Figure 6:**
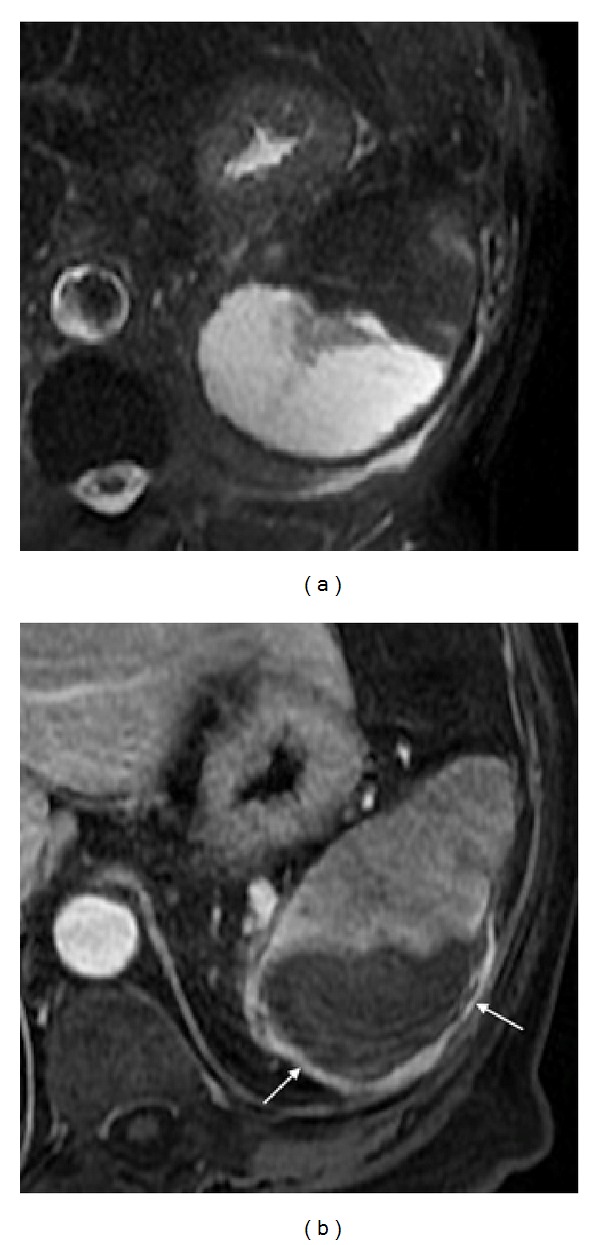
Splenic abscess. Axial T2wi SSFSE with fat suppression (a) and postcontrast axial T1wi 3D-GRE with fat suppression in the venous (b) phase. A large subcapsular splenic abscess is depicted. This lesion is marked hyperintense on T2wi and hypointense on T1wi with irregular margin. Note the faint enhancement of the splenic capsule (arrows, (b)).

Splenic abscesses are hypointense on T1-weighted images and have a moderate to high signal intensity on T2-weighted images, with irregular and undefined margins [8, 16]. Gas might be seen within the abscess as signal voids in the antidependent position and might be recognized by the presence of susceptibility artifact on T1-weighted in-phase and out-of-phase GRE sequences which appear greater on sequences with higher TE the sequence with higher TE (usually in-phase images). Following intravenous contrast administration, peripheral enhancement may be seen, although it is less often intense compared to liver abscess, perhaps due to the fairly bright enhancement of the normal splenic parenchyma in the arterial phase [8, 16] (Figure 6).

**Figure 7:**
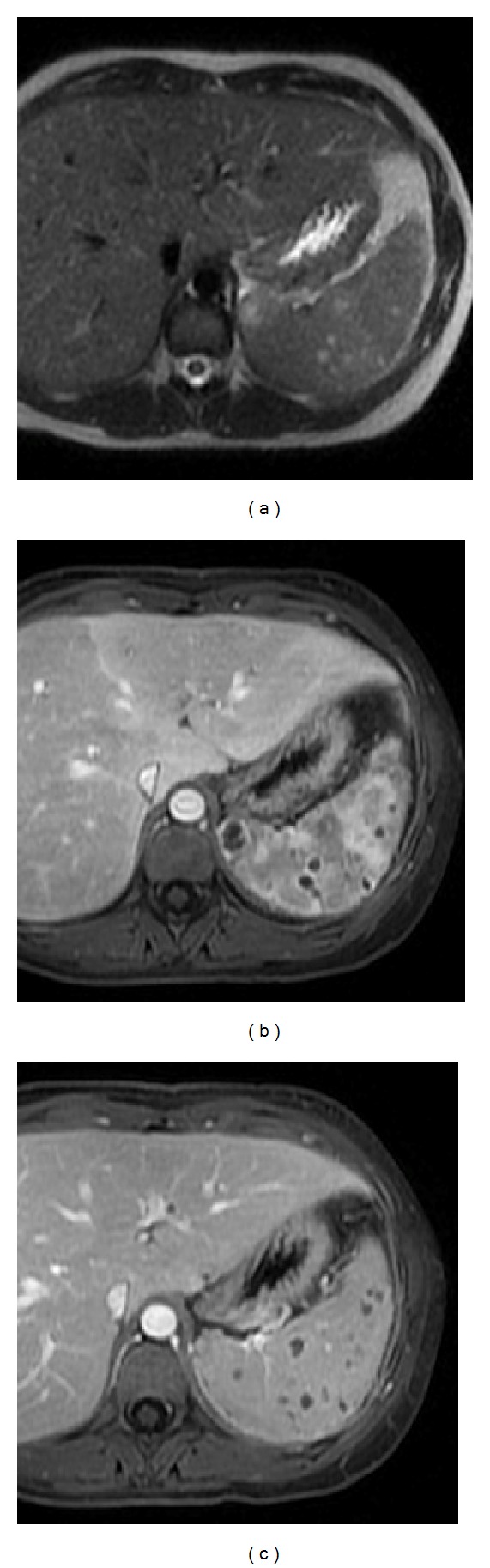
Microabscesses in an HIV patient with candidiasis. Axial T2wi SSFSE (a) and postcontrast axial 3D-GRE T1wi with fat suppression at the arterial (b) and venous (c) phases images. Multiple ill-defined T2w hyperintense lesions (a) with peripheral ring enhancement on gadolinium-enhanced images ((b) and (c)) are depicted, consistent with abscesses.

Candidiasis is the opportunistic infection that most frequently affects the liver and the spleen in immunocompromised patients. MRI is superior to CT for the detection and characterization of splenic microabscesses (<1 cm), most commonly secondary to candidiasis and appear as multiple hyperintense lesions in T2-weighted images with peripheral ring enhancement on gadolinium-enhanced images [7, 8] (Figure 7).

### 4.2. Histoplasmosis

Although it might be seen in patients with competent immune systems, the prevalence of histoplasmosis is greater in immunocompromised patients. MR imaging demonstrates the acute and subacute phases of this disease as scattered hypointense lesions on both T1- and T2-weighted images. Old granulomas can be calcified, causing with the characteristic signal intensity changes with blooming artifacts on MR images [8].

### 4.3. Sarcoidosis

Sarcoidosis is a granulomatous systemic disease of unknown etiology that may involve several organs and not infrequently the spleen. Of patients with systemic sarcoidosis, 24 to 59% have biopsy-documented splenic sarcoidosis [17].

**Figure 8:**
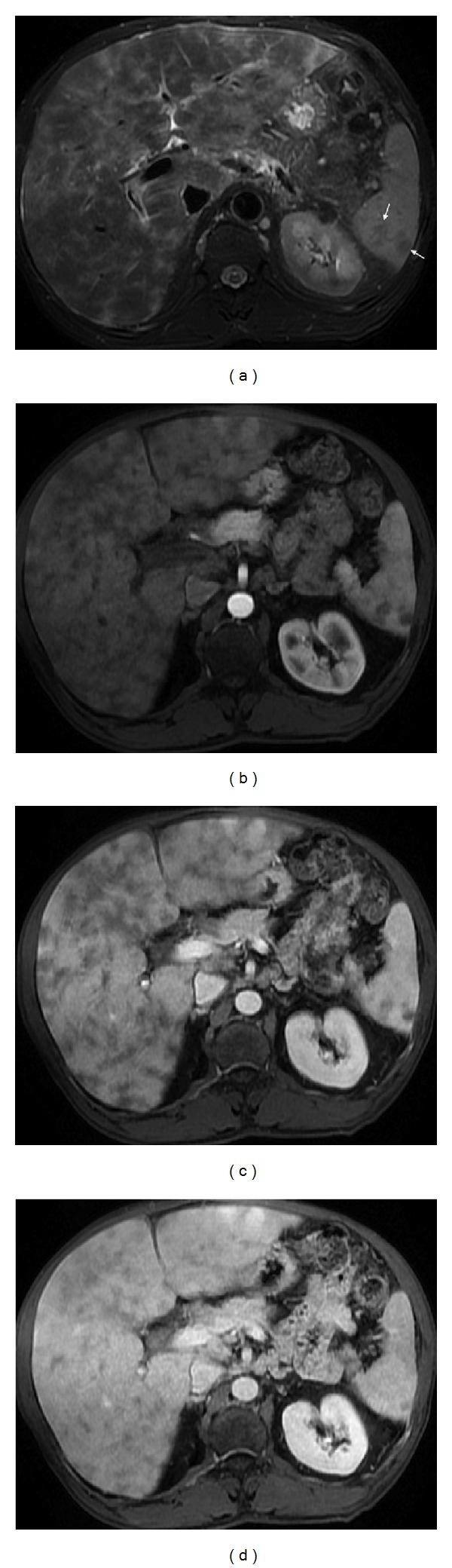
Sarcoidosis. Axial T2wi FSE with fat suppression (a) and postcontrast axial T1wi 3D-GRE with fat suppression in the arterial (b), venous (c) and interstitial phases (d). Low signal intensity nodules are depicted in the spleen on the T2wi sequences (arrows, (a)). The lesions are most conspicuous on fat-suppressed T2wi or early phase (b) contrast-enhanced images. Note the progressive enhancement of the sarcoid lesions on venous (c) and delayed images (d).

Nodular sarcoidosis has been reported to demonstrate low signal intensity in all MR sequences. The lesions are most conspicuous on T2-weighted fat-suppressed or early phase contrast-enhanced images. Sarcoid lesions enhance minimally on delayed images (Figure 8).

## 5. Vascular Lesions

### 5.1. Infarct

Splenic infarcts may result from venous or arterial blood supply interruption. The vascular occlusions can be the result of a tromboembolic process caused by any type of hemolytic anemia, endocartitis or chronic valvular diseases, Gaucher disease, portal hypertension, or vascular collagen diseases [8, 18].

**Figure 9:**
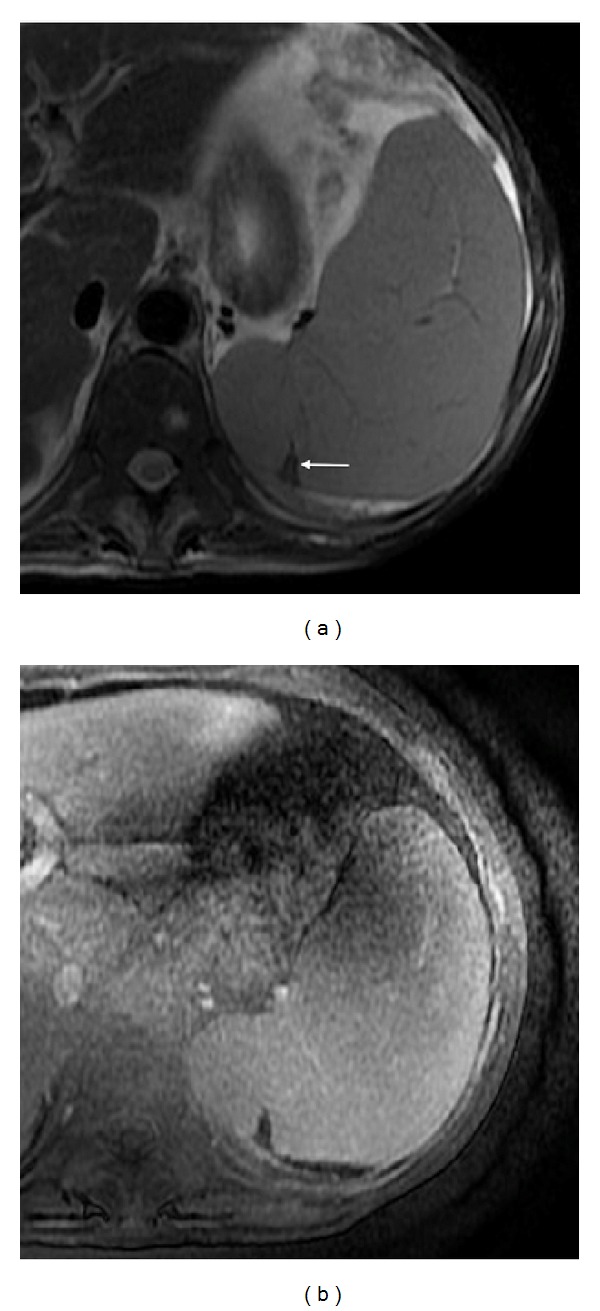
Splenic infarct. Axial T2wi FSE (a) and axial postcontrast fat-suppressed 3D-GRE T1wi at the venous (b) phase. A small triangular wedge-shaped area at the periphery of the spleen is noted (arrow, (a)), with hypointensity signal on both T1 and T2wi, with no enhancement on postcontrast images.

The typical MR appearance of a splenic infarct is a triangular wedge-shaped area, at the periphery of the spleen, with varying signal intensity according to the age of the infarct. It shows no enhancement after gadolinium injection and it is better depicted in late vascular phases, when the spleen is homogeneously enhanced [7, 8] (Figure 9).

### 5.2. Hematoma

**Figure 10:**
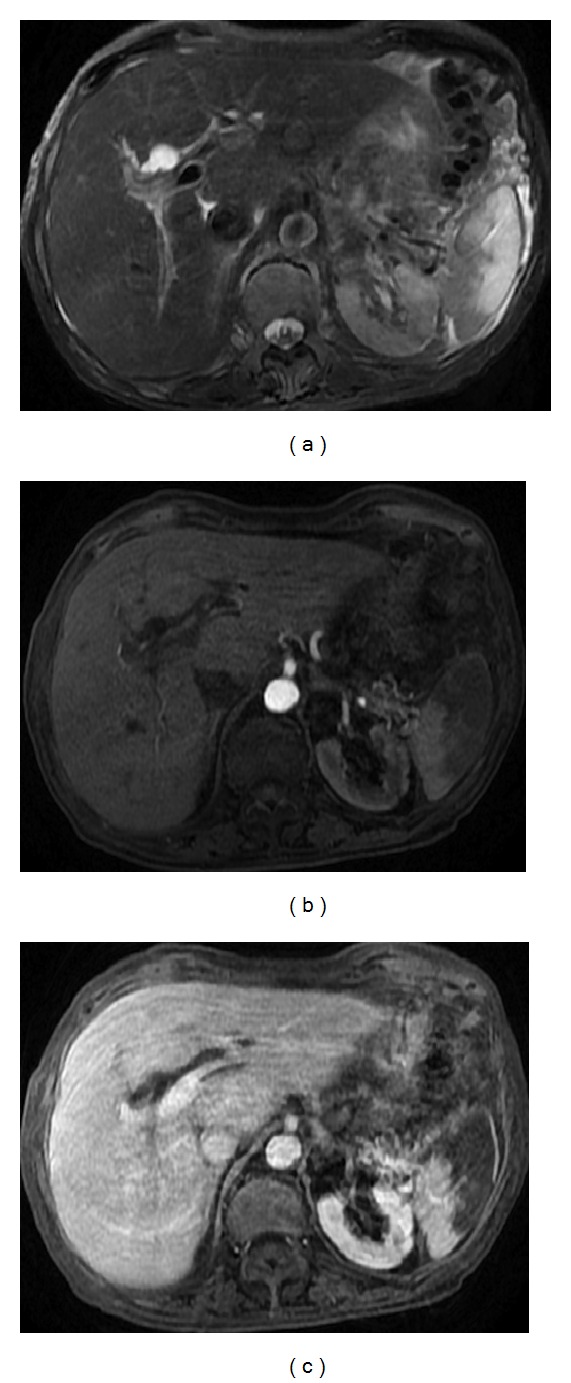
Hematoma. Axial (a) T2wi SSFSE and postcontrast axial T1wi 3D-GRE with fat suppression in the arterial (b) and interstitial (c) phases. A chronic hematoma is depicted with a cystic appearance, regarded as a lesion with moderate hyperintensity on T2wi sequences (a) and hypointensity on T1wi with no perceptible enhancement ((b), (c)).

Splenic hematoma is usually secondary to trauma [18]. Like splenic infarcts, the MR appearance is variable, depending on the age of the lesion. On acute (1 to 2 days) and early subacute phase (2 to 7 days) hematomas show low signal intensity on T2-weighted images and intermediate and increasingly higher signal intensity on T1-weighted images, respectively. On late subacute phase (7 to 14–28 days), hematomas show hyperintensity on both T1- and T2-weighted images (Figure 10). After 3 weeks (chronic), the hematoma may have a cystic appearance, regarded as a hyperintensity lesion on T2-weighted sequences with low signal intensity on T1-weighted images [18]. Older hematomas appear hypointense on both T1- and T2-weighted images, due to its fibrotic component.

## 6. Benign Tumors

### 6.1. Cysts

Cysts are the most common benign focal splenic masses. They may be separated into epithelial or true cysts (approximately 25% of splenic cysts), which are lined with epithelium, and pseudocysts that can be posttraumatic or secondary to pancreatitis, in which the wall is fibrotic and lacks a true cellular lining (account for approximately 75% of splenic cysts) [1, 19]. 

Posttraumatic pseudocysts are thought to represent the final stage in evolution of a splenic hematoma, although some have suggested that they might also be secondary to infarct or infection [16, 18]. Pancreatic pseudocysts arising in the tail of the pancreas may involve the spleen by extending either beneath the splenic capsule or into the proper splenic parenchyma. Patients usually have a history of acute pancreatitis [16].

**Figure 11:**
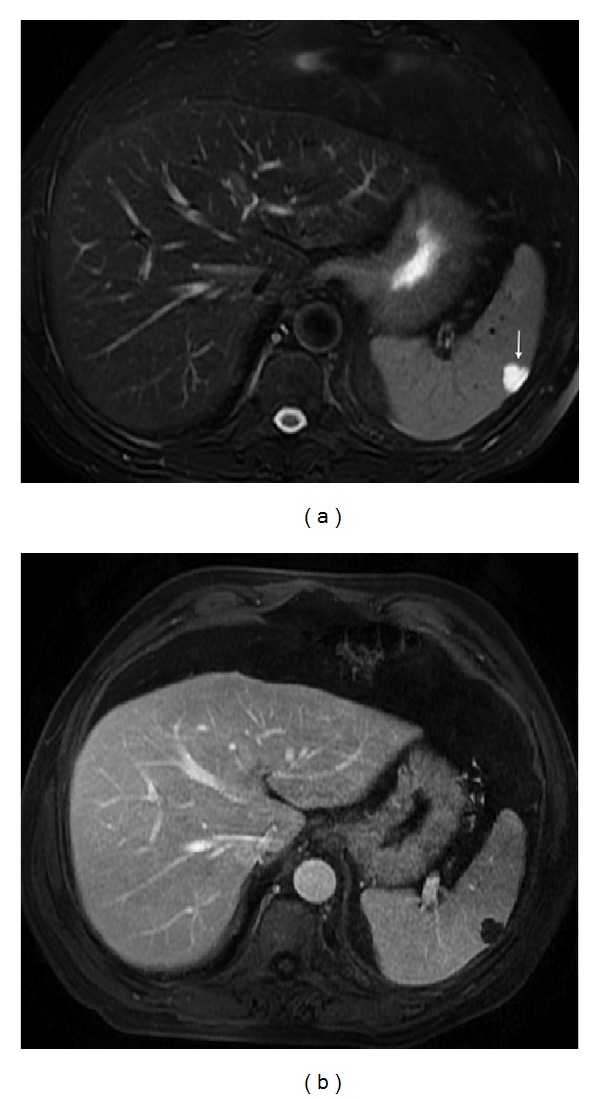
Splenic cyst. Axial T2wi FSE with fat suppression (a) and postcontrast axial 3D-GRE T1wi with fat suppression at the interstitial (b) phase. Note the thin-walled and well-defined nodule, homogeneously hyperintense on T2wi (a), with no enhancement on post contrast image, characteristic of cysts.

MRI shows the characteristic findings of a cyst, that is, a well-defined round mass, with thin or imperceptible wall, homogeneous hypointense on T1-weighted images, and hyperintense on T2-weighted images, with no enhancement on postcontrast images [18] (Figure 11).

It is often impossible to distinguish between true and false cysts; the clinical presentation and patient history may help to narrow the differential diagnoses. Cyst wall trabeculation or peripheral septations are much more commonly found in true cysts [7].

### 6.2. Hydatid Cyst (*Echinococcus granulosus*)

**Figure 12:**
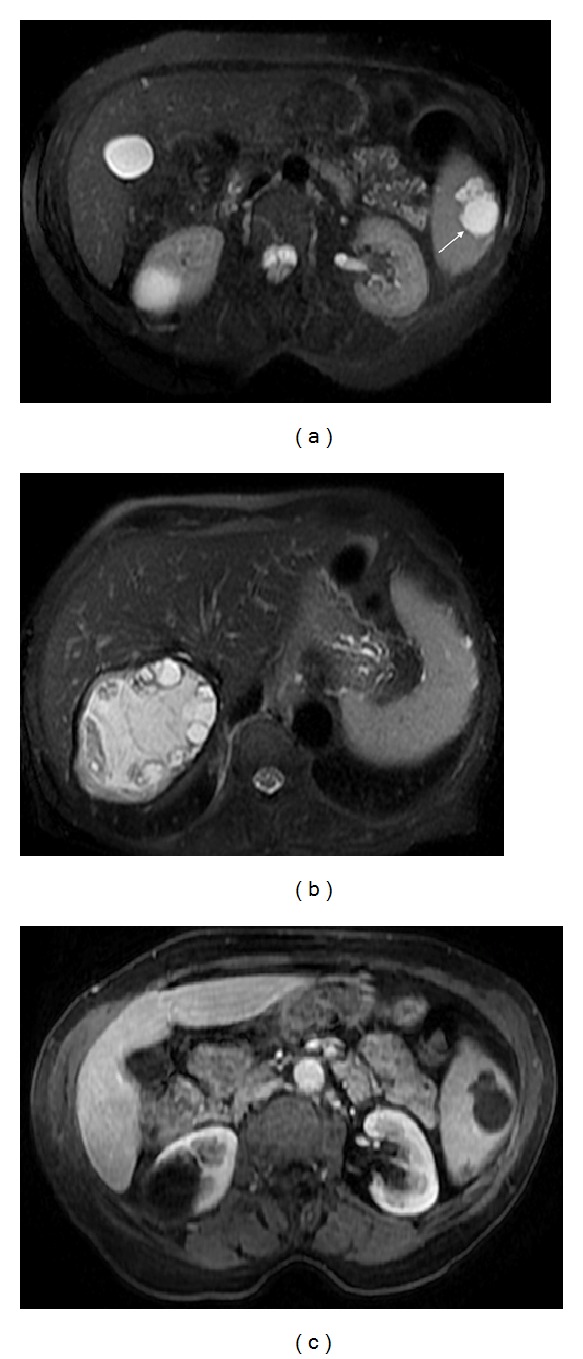
Hydatid cyst. Axial T2wi SSFSE with fat suppression ((a) and (b)) and axial postcontrast T1wi 3D-GRE with fat suppression in the venous (c) phase. A classic hydatid cyst is visualized in the liver (b). A concomitant splenic hydatid cyst is depicted as a multilocular lesion with moderate hyperintensity on T2wi (arrow, (a)) and hypointensity on T1wi (c). A fibrotic thickened continuous low signal intensity rim surrounding the cyst is seen on T2wi ((a), (b)). No enhancement is noted following IV contrast administration (c).

Usually involves the liver or lungs but occasionally may also involve the spleen. Splenic hydatid cysts are very rare even in endemic regions (less than 2%) [19]. On MRI these cysts share imaging characteristics to those located in the liver. They are hyperintense on T2-weighted images and hypointense on T1-weighted images. They can be either unilocular or contain daughter cysts, distributed peripherally or throughout the lesion, giving a multilocular appearance. A “serpent” or “snake” sign is occasionally noted representing collapsed parasitic membranes within the cyst. Continuous and irregular, 4- to 5-mm-thick, low signal intensity rim surrounding the cyst corresponding to the dense fibrous capsule encasing the parasitic membranes is frequently seen. Typically, no enhancement is noted following IV contrast administration [18, 20] (Figure 12).

### 6.3. Hemangiomas

Splenic hemangiomas are the most common benign solid tumor of the spleen. Their frequency in large autopsy series is 0.03%–14%, and they are more frequently found in males (1.4 versus 1.0) [19, 21]. These lesions are believed to be congenital in origin, arising from sinusoidal epithelium. Most of them are less than 2 cm in diameter; however, once large they may spontaneously rupture, causing intra-abdominal hemorrhage. Histologically, they are composed of endothelial-lined blood-filled spaces of varying size and can be characterized by the size of these spaces as capillary or cavernous lesions [16].

Diffuse hemangiomatosis of the spleen is a rare benign vascular condition occurring as a manifestation of systemic angiomatosis (associations with Klippel-Trénaunay-Weber, Turner, Kasabach-Merritt-like, and Beckwith-Wiedemann syndromes have been reported) or, less commonly, confined to the spleen [8].

**Figure 13:**
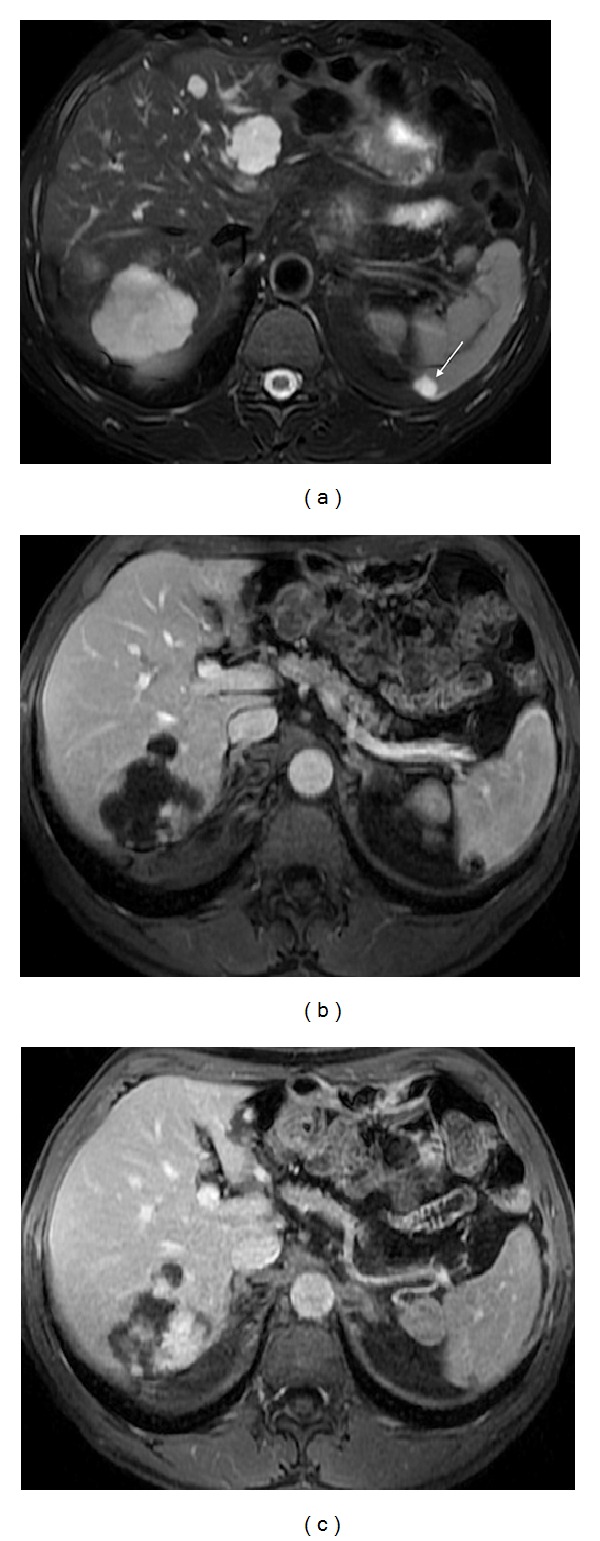
Splenic hemangioma. Axial T2wi FSE with fat suppression (a) and postcontrast axial 3D-GRE T1wi with fat suppression at the arterial (b) and interstitial (c) phases. The hemangioma is depicted as a well-defined, homogeneous, and hyperintense lesion on T2wi (arrow, (a)), with a peripheral enhancement with centripetal and delayed progression, on postcontrast images ((b) and (c)). Note the hepatic hemangiomas on the same imaging plane.

Most hemangiomas are well-defined homogeneous, hypo- to isointense on T1-weighted images and most commonly hyperintense on T2-weighted images compared with splenic parenchyma [21] (Figure 13). On dynamic contrast-enhanced studies, they usually show peripheral enhancement with centripetal, delayed progression (see Figure 13). Uncommonly, similarly to liver hemangiomas, these lesions may undergo sclerosis and late phase images are important to suggest the correct diagnosis. The typical nodular peripheral enhancement of hepatic hemangiomas is uncommonly seen in splenic hemangiomas [22].

### 6.4. Hamartomas

Hamartoma is an infrequently benign, usually asymptomatic tumor of the spleen, with an autopsy incidence of 0.13% and no gender predilection. Approximately, one-sixth of hamartomas are found in children (<16 years). They are nonneoplastic tumors composed of a mixture of normal elements of splenic red and white pulp components [19, 23]. They are considered to be congenital in origin, but some theories associate it with previous trauma.

**Figure 14:**
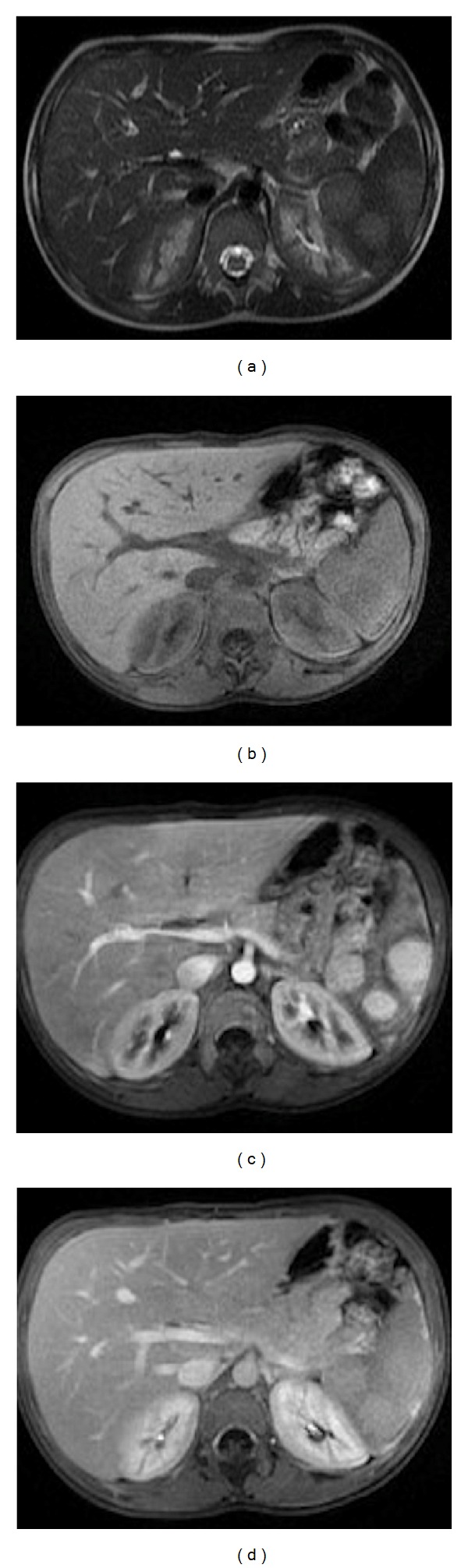
Splenic hamartoma. Axial T2wi SSFSE (a), pre- (b) and postcontrast axial T1w 3D-GRE with fat suppression in the arterial (c) and venous (d) phase. Multiple rounded lesions are seen on T2wi (a) and T1wi (b). These lesions demonstrate hyperenhancing characteristics on the arterial phase (c) progressing to isointensity on the venous phase (d).

Hamartomas are a sharply defined, rounded, single solid lesion that sometimes may be multiple (Figure 14). On MRI, they are usually isointense on T1-weighted images and heterogeneously hyperintense on T2-weighted images [22]. After intravenous gadolinium administration, there is usually diffuse heterogeneous enhancement, which may be useful in distinguishing this lesion from the typical peripheral enhancement noted in hemangiomas (see Figure 14). Prolonged enhancement may be appreciated, which has been attributed to stagnant contrast material within the sinusoids of the red pulp of splenic tissue. Persistent areas of hypointensity may also be seen and correspond to areas of necrosis within the lesion [7, 16, 21].

### 6.5. Littoral Cell Angioma

Littoral cell angioma is a relatively new clinicopathological entity of a rare benign tumor of the spleen that develops from the lining cells of the red-pulp sinuses, the so-called “littoral cells”, giving rise to littoral cell angioma [24]. It has no malignant histological features and has a benign clinical course.

Lesions are of variable size and commonly multinodular. They are composed of anastomosing vascular channels with irregular lumina featuring cyst-like spaces and lined by tall endothelial cells. 

**Figure 15:**
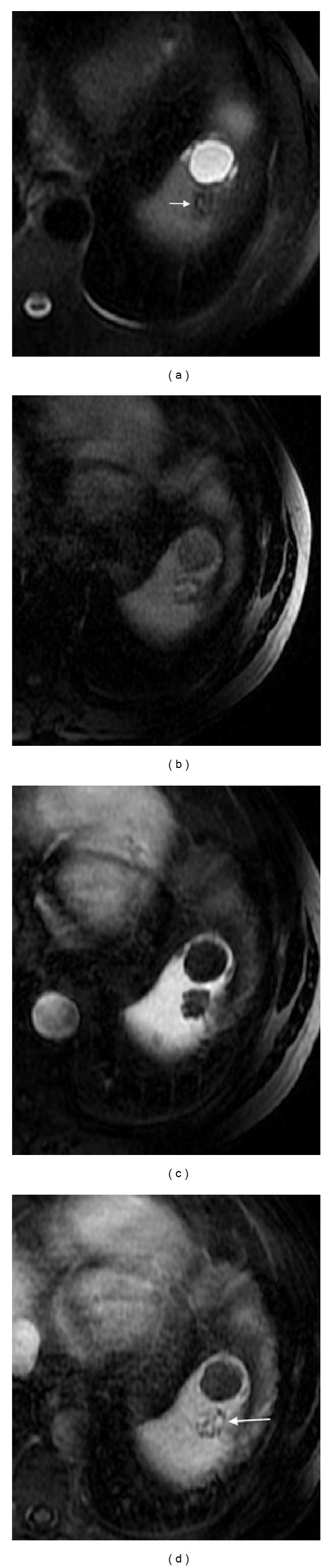
Littoral cell angioma. Axial T2wi FSE with fat suppression (a), pre- (b) and postcontrast axial 3D-GRE T1wi with fat suppression at the venous phase (c) and after 10 minutes of contrast injection (d). A hypointense nodular lesion is depicted on T2wi (arrow, (a)), with areas of magnetic susceptibility artifact, and hypovascular nodules that show subtle peripheral enhancement with progressive slow centripetal accumulation of contrast (arrow, (d)). This mass was thought to represent a sclerosed splenic hemangioma. This heterogeneous splenic appearance is also possible with angiosarcoma and in cases of splenic hemangiomatosis. Note the anteriorly adjacent splenic cyst.

On MRI, lesions are inhomogeneously hyperintense on T2-weighted images, with signal intensity similar to that of hemangiomas and slightly hypointense on unenhanced T1-weighted images. Littoral cell angiomas may show low signal intensity on all sequences due to hemosiderin accumulation within neoplastic littoral cells [25]. Dynamic postcontrast T1-weighted images depict delayed contrast enhancement, suggestive of a vascular lesion with contrast media pooling (Figure 15).

### 6.6. Lymphangioma

Lymphangioma is a rare vascular benign lesion filled with lymph instead of red blood cells as seen in hemangioma [18]. Usually diagnosed in childhood, it may appear as solitary or multiple splenic lesions or as a diffuse involvement replacing most of the splenic parenchyma, known as lymphangiomatosis [16].

Cystic lymphangioma is the most frequent type and is characterized by a honeycomb of large and small thin-walled cysts containing lymph-like clear fluid. On MRI, lymphangiomas usually present as well-defined multilocular cystic lesions, with thin septations and hyperintensity on T2-weighted sequences. However, some of the cysts may be hyperintense on T1-weighted images, due to protein or hemorrhagic content [1, 16, 19].

## 7. Malignant Tumors

### 7.1. Lymphoma

Lymphoma is the most common splenic malignancy. Both Hodgkin's and non-Hodgkin's lymphoma may present in the spleen as the primary site (less than 1%) or as part of systemic involvement [1].

Splenic involvement in lymphoma may produce homogeneous enlargement (the most common finding, although it may be absent in up to 30% of patients), multiple small (or miliary) nodules, a single solitary mass, or a combination of these appearances [16, 19].

Immediate postcontrast MRI images surpass CT in their evaluation; nevertheless, the role of MRI has not been established yet.

**Figure 16:**
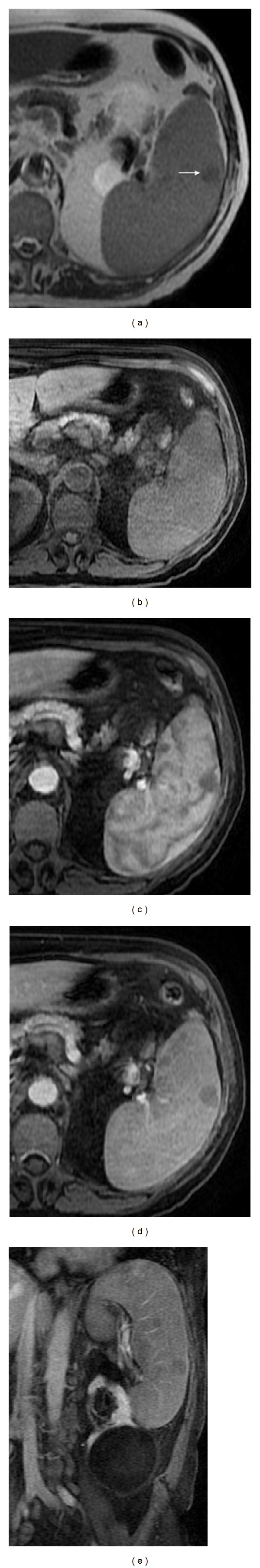
Lymphoma. Axial T2wi SSFSE (a), axial pre- (b) and postcontrast T1wi 3D-GRE with fat suppression in the arterial (c) and venous (d) phase. Coronal fat-suppressed T1wi 3D-GRE in the interstitial phase (e). The spleen is enlarged. Lymphomatous nodules are isointense to splenic parenchyma on T1wi (b) and T2wi (a). One nodule is moderately hypointense T2wi (arrow, (a)). This feature aids in distinction against metastatic lesions, which are commonly hyperintense. Lymphomatous lesions demonstrate hypovascular nature with lower signal intensity relative to normal spleen on postcontrast images ((c), (d) (e)), thereby increasing conspicuity.

Lymphomatous nodules are typically isointense to splenic parenchyma on T1- and T2-weighted images, although they may present some hypointensity on T2-weighted images, which may help to distinguish from metastatic lesions (Figure 16). Lymphomatous lesions are usually hypovascular with lower signal intensity relative to normal spleen on postcontrast images, thereby increasing conspicuity [16, 23].

### 7.2. Metastasis

Although the spleen is the most vascular organ in the body, it is an infrequent site for metastatic disease. Metastatic involvement of the spleen is somewhat uncommon, occurring in up to 7% of patients with widespread malignancy. According to most series, splenic metastases are mainly due to melanoma and breast cancers and in a less percentage from cancers of the lung, colon, stomach, ovary, endometrium, and prostate [7, 18].

**Figure 17:**
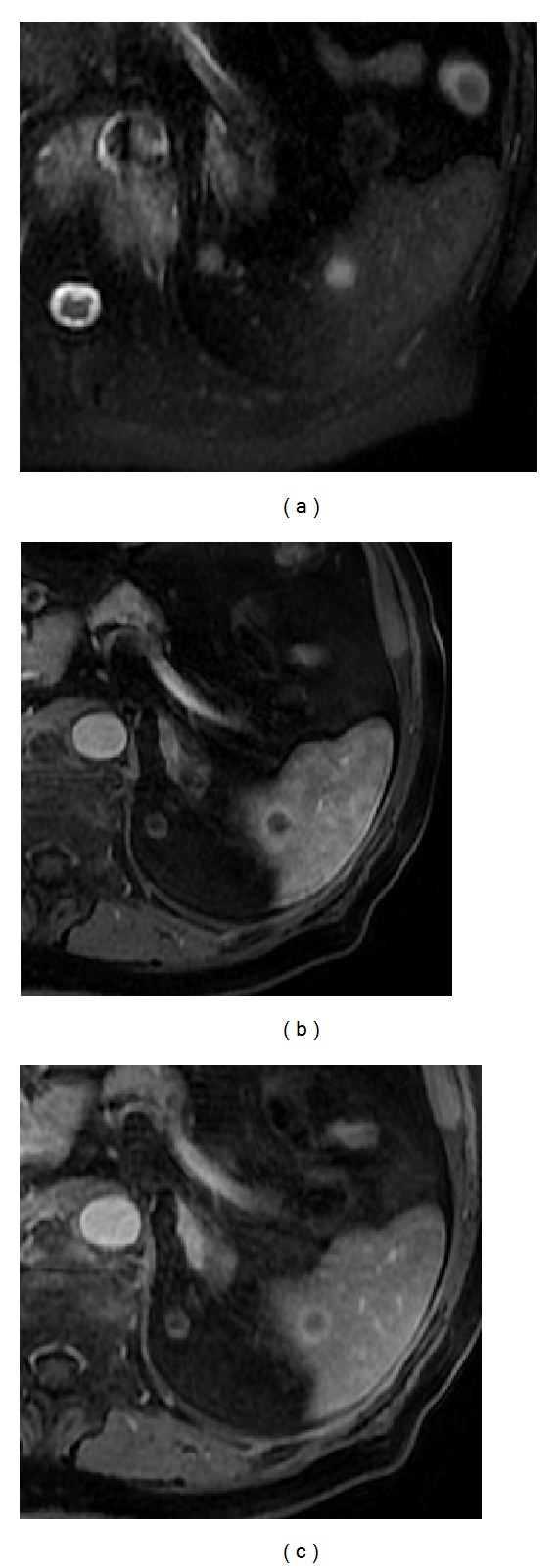
Splenic metastasis on a patient with a small-cell lung carcinoma. Axial T2wi SSFSE (a) and postcontrast axial 3D-GRE T1wi with fat suppression at the arterial (b) and venous (c) phases. Note the nodular lesion depicted as a hyperintense nodule on T2wi with peripheral ring-like enhancement.

Splenic metastatic lesions may be solitary, multiple, or diffuse and differ in number and size from a few millimeters to several centimeters. At MR imaging, metastases typically appear as hyperintense masses on T2-weighted images and hypo- to isointense masses on T1-weighted images with inhomogeneous contrast enhancement, usually with peripheral ring-like pattern [19, 23, 26] (Figure 17). Central tumor necrosis is seen as regions of hyperintensity on T2-weighted images [27]. The presence of blood products from hemorrhage or other paramagnetic substances, such as melanin from melanocytic melanomas, may result in high signal intensity on T1-weighted images [18].

### 7.3. Perisplenic Neoplasms Infiltrating the Spleen

**Figure 18:**
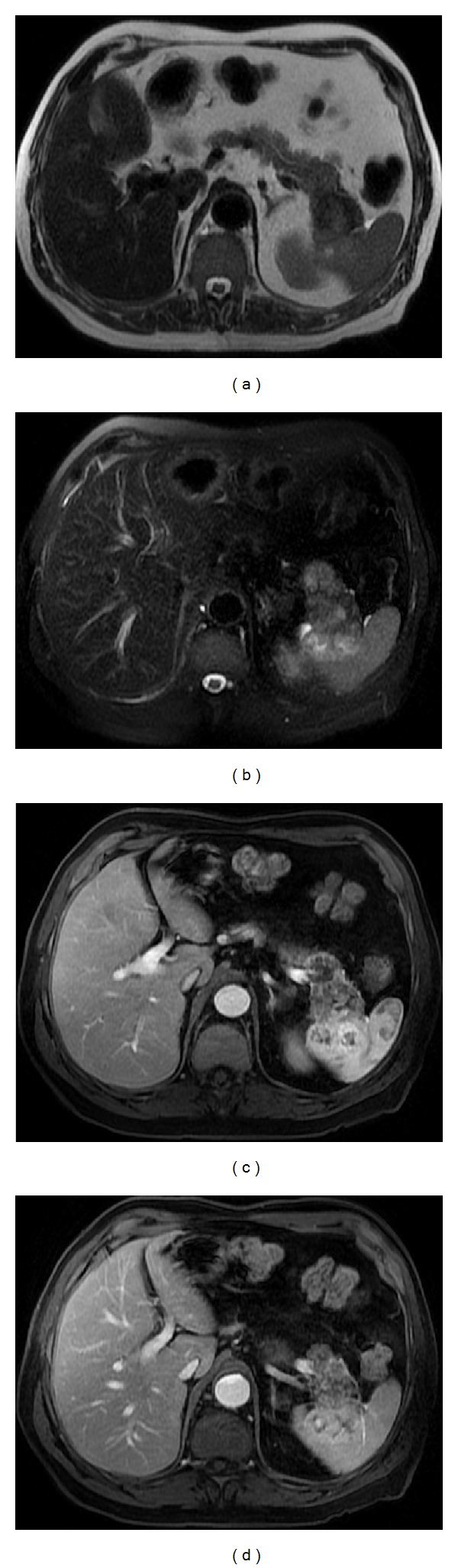
Pancreatic tail clear cell renal cell carcinoma metastases with infiltration of the spleen through the splenic hilum. Axial T2wi SSFSE without (a) and with (b) fat suppression and axial postcontrast T1wi 3D-GRE with fat suppression in the arterial (c) and venous (d) phase. A large heterogeneous mass is seen in the pancreatic tail infiltrating the spleen.

Implants on the serosal surface of the spleen are seen in patients with peritoneal carcinomatosis, commonly from ovarian or gastrointestinal primary neoplasms. These implants may cause indentation and scalloping of the surface of the spleen. Direct tumor invasion of the spleen is uncommon, but can be seen in tumors originating from the pancreas, stomach, colon or left kidney, and retroperitoneum (Figure 18) [28].

### 7.4. Angiosarcoma

Angiosarcoma is exceedingly rare, yet it is the most common primary malignant nonlymphoid tumor of the spleen [29]. These tumors are highly aggressive (nearly 80% of patients die 6 months after the diagnosis) and usually manifest as widespread metastatic disease or splenic rupture [7, 8, 16]. Association with thoratrast has been reported.

Angiosarcoma typically appears as multiple nodular heterogeneous masses, with variable signal intensity on T1-weighted and T2-weighted images, due to the presence of hemorrhage with different ages, siderotic nodules, and areas of necrosis. Following the intravenous administration of gadolinium, the lesion demonstrates heterogeneous enhancement. MRI seems to be more precise than CT in the overall assessment and staging of this type of tumor and is of particular value for timely diagnosis [30].

There are other extremely rare primary malignant splenic tumors including malignant fibrous histiocytoma, leiomyosarcoma, fibrosarcoma, malignant teratoma, and Kaposi sarcoma, all of which are with nonspecific appearance.

## 8. Diffuse Diseases

### 8.1. Splenomegaly

**Figure 19:**
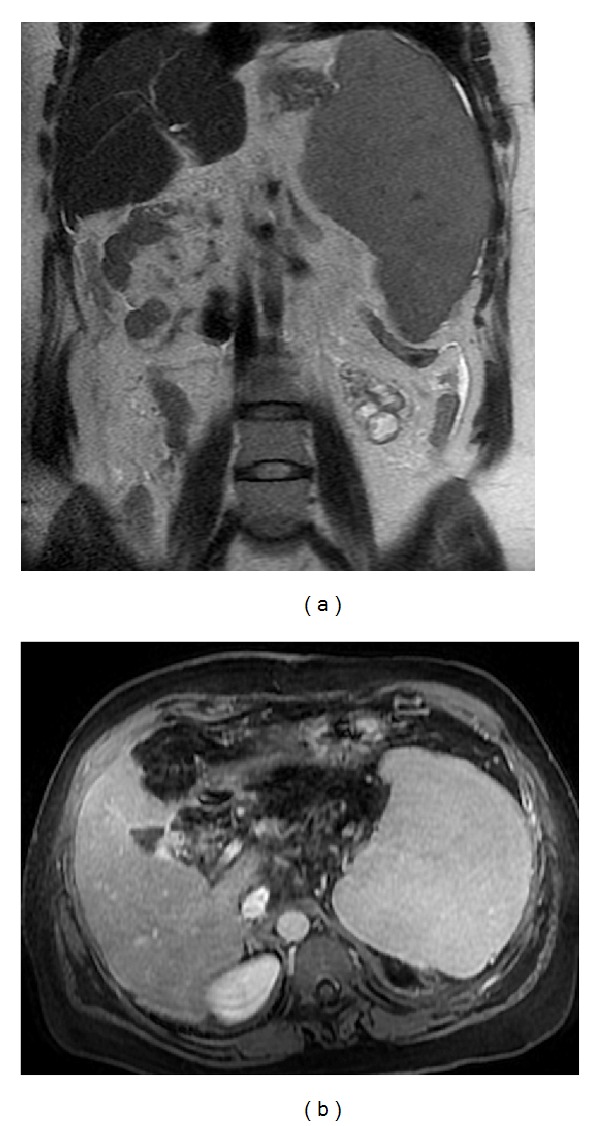
Splenomegaly. Coronal T2wi SSFSE (a) and postcontrast axial T1wi 3D-GRE with fat suppression in the interstitial phase (b). A homogeneous splenomegaly resulting from congestion (portal hypertension) is easily seen.

Splenomegaly is a radiologic and clinical sign, classically described when the craniocaudal splenic length is more than 12 cm (Figure 19). This may result from congestion (portal hypertension, splenic vein occlusion, or thrombosis), infiltrative disease (Gaucher disease or histiocytosis), hematologic disorders (polycythemia vera, myelofibrosis), inflammatory's/infectious diseases (HIV, mononucleosis, amyloidosis, Feltys syndrome, or mycobacterial infection), cysts, or tumors (leukemia, lymphoma, or metastases) [7, 10, 23].

### 8.2. Siderotic Nodules

**Figure 20:**
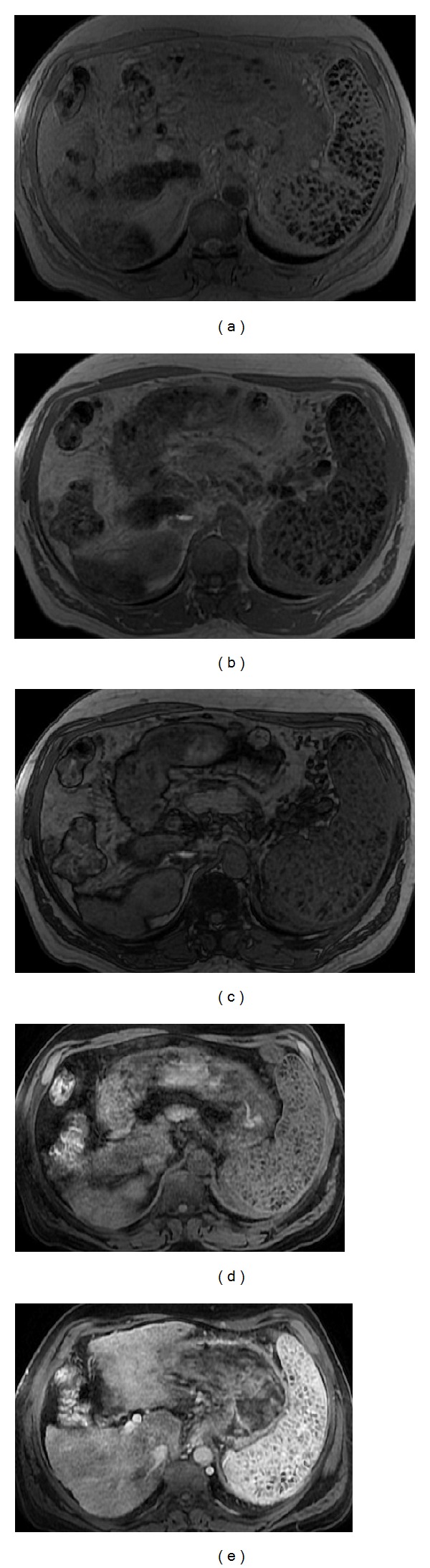
Siderotic splenic nodules (Gamma-Gandy bodies). Axial T2* (a), axial 2D-GRE T1w in-phase (b) and out-of-phase (c), and pre (d) and postcontrast axial 3D-GRE T1wi with fat suppression at the arterial phase (e). Note the splenomegaly with multiple foci of hemosiderin with low signal intensity on all pulse sequences and exhibiting the “blooming” artifact on in-phase (longer TE) GRE sequences, secondary to iron deposition. No enhancement is depicted.

Foci of hemosiderin deposition are seen in about 9%–12% of patients with portal hypertension and are the so-called Gamma-Gandy bodies (Figure 20). These foci of hemosiderin have low signal intensity on all pulse sequences and exhibit “blooming” artifact on gradient echo sequences, secondary to iron deposition [8, 23].

### 8.3. Gaucher Disease

Gaucher disease is an autosomal recessive lysosomal disorder secondary to lack of the enzyme glucocerebrosidase, leading to the accumulation of glucocerebrosides in the cells of the reticuloendothelial system, causing hepatosplenomegaly. Splenic infarcts and fibrosis associated with Gaucher disease may exhibit a multifocal pattern [8].

### 8.4. Hemosiderosis and Sickle Cell Disease

**Figure 21:**
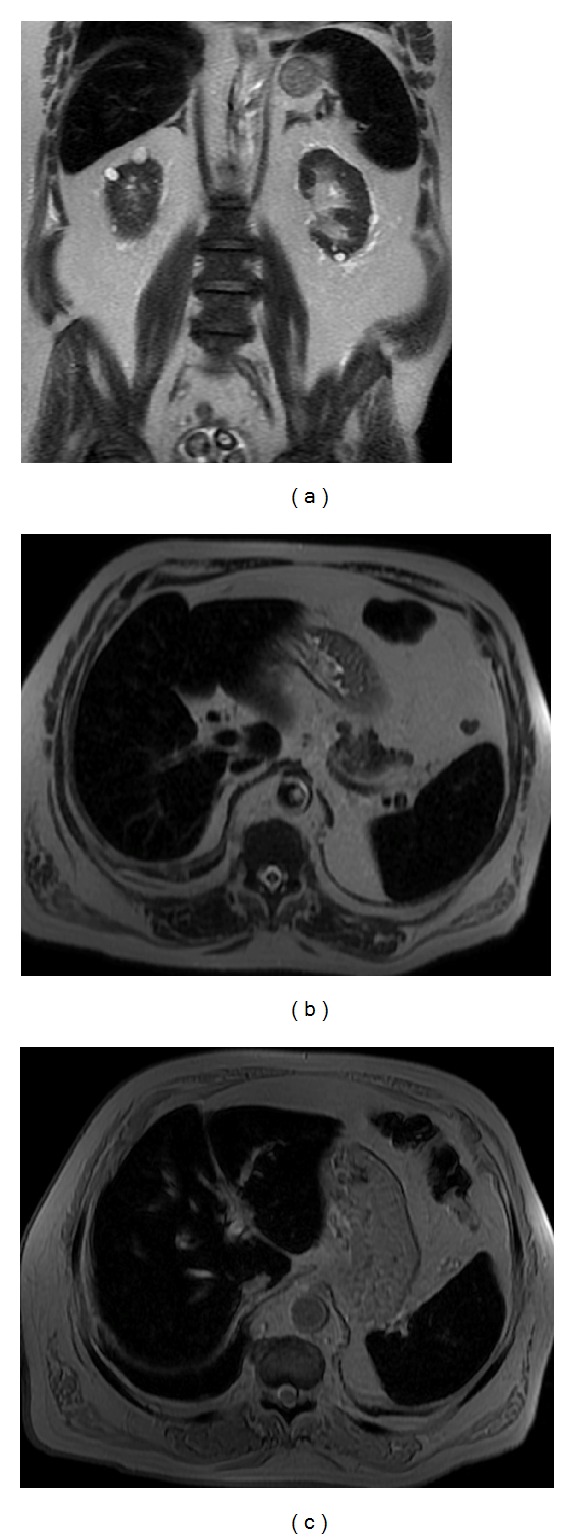
Paroxysmal nocturnal hemoglobinuria. Coronal (a) and axial (b) T2wi SSFSE and axial T2* (c) images. This patient with paroxysmal nocturnal hemoglobinuria shows diffuse diminished signal intensity of the liver and spleen on T2wi as a result of hemosiderin deposition. Notice the iron accumulation on the renal cortex (a).

Hemosiderosis, with splenic involvement, shows diffuse diminished signal intensity of the organ on both T1- and T2-weighted images relative to musculature as a result of hemosiderin deposition [1] (Figure 21).

**Figure 22:**
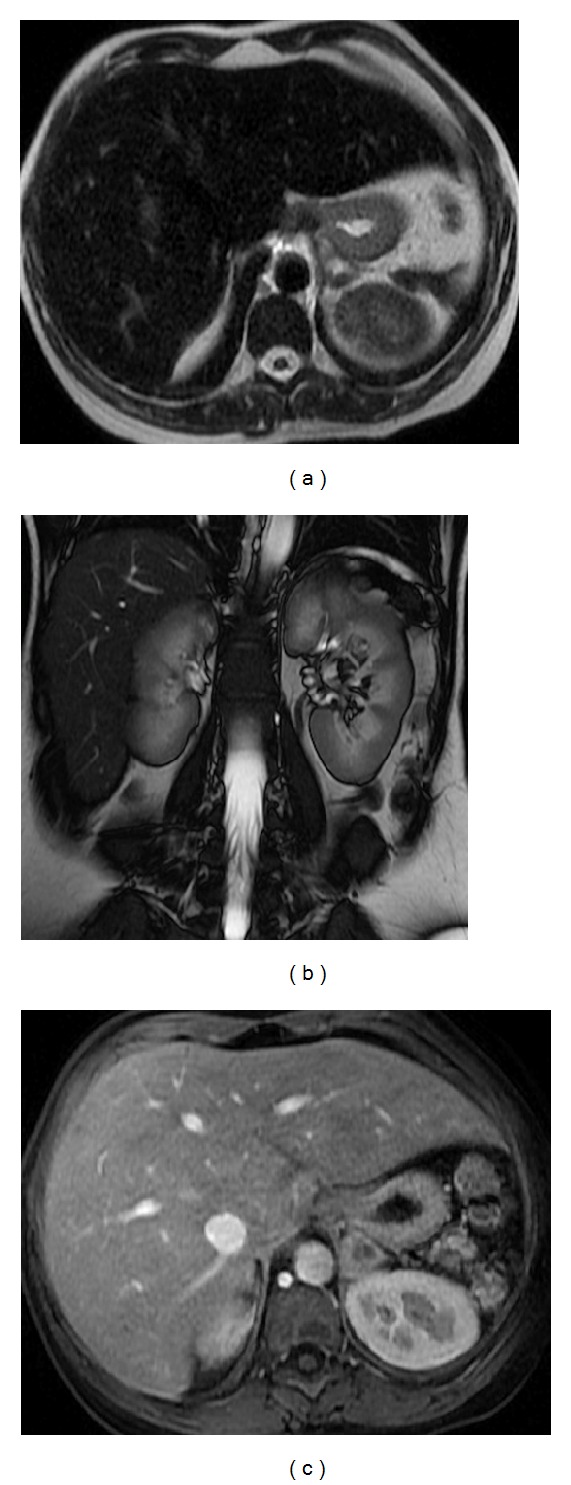
Autosplenectomy is found in a patient with homozygous sickle cell disease. Axial T2wi SSFSE (a), coronal SSFP (b), and postcontrast axial 3D-GRE T1wi with fat suppression at the arterial phase (c). Note the small remnant of spleen and the diffuse diminished signal intensity of the hepatic parenchyma on both T1wi and T2wi as a result of iron deposition.

Sickle cell disease is common in the Afro-descendent population with a prevalence of 0.2% (homozygous form) and 8%–10% (heterozygous form). The spleen is the most commonly organ involved. In patients with sickle cell disease, the spleen appears as a nearly signal void area due to iron deposition from blood transfusion. Autosplenectomy is often found in patients with homozygous sickle cell disease [7, 8] (Figure 22).

### 8.5. Extramedullary Hematopoiesis

Extramedullary hematopoiesis is a compensatory response to failure of the bone marrow cells. A focal mass-like involvement of the liver and spleen, which are the main affected organs, may be present. On MRI, the appearance of the nodular lesions depends on the evolution of the hematopoiesis. Active lesions reveal intermediate signal intensity on T1-weighted images, high signal intensity on T2-weighted images, and moderate enhancement after administration of intravenous gadolinium. Older lesions are hypointense on T1- and T2-weighted images and do not show any enhancement. These lesions usually demonstrate low signal intensity on in-phase T1-weighted GRE images compared with that on out-of phase images due to the presence of iron [8].

## 9. Conclusion

Focal and diffuse lesions of the spleen are uncommon and usually discovered incidentally on cross-sectional imaging studies. Due to the widespread of MRI there has been an increase in the detection of diseases involving the spleen. By virtue of its excellent contrast resolution and the possibility of tissue characterization through the use of different sequences, MRI provides an excellent tool for the evaluation and characterization of various splenic lesions. 

Awareness of the MRI appearance of the most common splenic disease processes is important for the radiologist to interpret the findings correctly, reaching whenever possible the appropriate diagnosis.
